# AMH measurement is being overused—Are you guilty?

**DOI:** 10.1111/aogs.70377

**Published:** 2026-10-19

**Authors:** Antti Perheentupa

**Affiliations:** ^1^ Ovumia Group Helsinki Finland; ^2^ Department of Obstetrics and Gynecology University of Turku Turku Finland; ^3^ Department of Obstetrics and Gynecology Turku University Central Hospital Turku Finland

In the recent number of *Fertility and Sterility*, de Ziegler et al. raise a very important concern about the widespread use, even suggestions of universal screening, of anti‐Müllerian hormone (AMH) levels in young women with no infertility.^1^ It is important to highlight the limitations and problems associated with AMH measurements as the use of this “diagnostic fertility tool” is gaining popularity in the Nordic countries and elsewhere.

AMH is secreted, not only by the granulosa cells surrounding the oocytes in small antral follicles, but also by those of preantral follicles. The circulating AMH concentration usually correlates fairly well with the antral follicle count (AFC). The number of antral follicles (2–9 mm in diameter) can be counted using transvaginal ultrasound. Both AFC and AMH concentration (i.e., ovarian reserve) can be used to estimate the ovarian response to gonadotropin stimulation in fertility treatments, most commonly IVF treatments. These parameters constitute ovarian reserve, which has found a routine place in the evaluation of women infertility and prior to planning the fertility treatment's details. The AMH concentration has also been proposed to be a biochemical marker for polyendocrine metabolic ovary syndrome (PMOS), also known as polycystic ovary syndrome (PCOS) until recently. Women with high as well as those with poor ovarian response can be identified with these tools. This is particularly important in avoiding ovarian hyperstimulation syndrome (OHSS) in women with PMOS. Combining the AMH level with the BMI has even enabled the use of algorithms in efforts to develop safe and efficient IVF stimulation to ensure optimal oocyte yield.^2^

The measurement of AMH concentration has spread from the above‐described appropriate use within treatment of infertility to routine measurement and evaluation of female fertility. At the moment it is obvious that AMH measurement is being overused, and often inappropriately interpreted in patient counseling. AMH concentration has no predictive value in evaluating the spontaneous fertility of women. The ovarian reserve (AMH and AFC) and oocyte quality as well as fertility decline with age. Hence, the reference levels for AMH concentration are age dependent, the reference range decreasing from 1.2–11.7 to 0.03–5.5 μg/L in 25‐ to 29‐year and 40‐ to 44‐old‐women, respectively.^3^ Furthermore, whereas the menstrual cycle phase has little effect on the AMH levels, particularly combined oral contraceptives are known to decrease the levels.^4^ It is important to understand that ovarian reserve and oocyte quality are independent. Removal of one ovary will dramatically decrease the ovarian reserve, but in young women the oocyte quality is not compromised, and spontaneous fertility is not significantly affected.

In evaluating the time to pregnancy (TTP), there was no significant difference in the lowest quintile compared to quintiles 2–4. It was the fifth quintile (highest AMH levels) that showed the longest time to pregnancy.^5^ This may be caused by an irregular or completely lacking menstrual cycle associated with an anovulatory cycle, which could well be a consequence of polyendocrine metabolic ovarian syndrome (PMOS—also known as polycystic ovary syndrome (PCOS) until recently). Elevated AMH levels have been suggested for identifying PMOS, where AMH levels could be used instead of evaluating the ovarian morphology by ultrasound.^6^ It is clear that women with high AMH levels will respond highly to gonadotropin stimulation and should be identified so that appropriate measures are taken to avoid OHSS. Most of these women are likely to have PMOS.

It has been proposed that low ovarian reserve would correlate with reduced euploidy rates in embryos obtained in IVF treatments. The recent large cohort study including 13 451 IVF cycles with pre‐implantation genetic testing (PGT‐A) showed that the low ovarian reserve, as evaluated by AMH and AFC, was not independently predictive of embryo euploidy.^7^ So while AMH levels can be appropriately used in estimating the ovarian response to the gonadotropin stimulation in IVF treatment, we should refrain from suggesting that low levels of AMH are associated with high rates of aneuploidy (nor that high levels of AMH are suggestive of high rates of euploidy). The euploidy rates are very clearly associated with female age irrespective of AMH levels.

Efforts to predict the time of menopause by AMH measurements have been challenging and the use of AMH to predict the age of spontaneous menopause remains imprecise.^8^ This may be explained by the rebound of AMH levels, which has been observed in more than half of women whose AMH levels had already diminished below the limit of detection. In a significant proportion of these women the rebound was associated with a concurrent rise in estradiol reflecting resurgence of ovarian function.^9^ Even unmeasurably low levels of AMH are no guarantee of lost fertility. Pregnancies have been reported in women with AMH levels <0.75 ng/mL, but also even with AMH levels <0.4 ng/mL.^10^, ^11^ It is therefore clear that discontinuation of contraceptive use should not be based on low AMH levels alone.

Appropriate fertility counseling should be available for couples and individuals. Sufficient knowledge, particularly on the effect of age and lifestyle choices on the likelihood of pregnancy and risk of miscarriage, needs to be relayed to the public, and preferably all health care professionals should have the basic knowledge of these facts. However, the widespread practice of largely basing the counseling on measured AMH levels should be avoided. Whereas, in planning the fertility treatments for patients with infertility, AMH has a clear role; it has little relevance in estimating the likelihood of a spontaneous pregnancy in women with no fertility issues. False interpretation of the levels in healthy women of fertile age can lead to significant anxiety as well as unnecessary procedures and treatments.

## AUTHOR CONTRIBUTION

Antti Perheentupa wrote the article.

## Data Availability

Data sharing not applicable to this article as no datasets were generated or analysed during the current study.
